# Fuzzy TOPSIS framework for promoting win–win project procurement negotiations

**DOI:** 10.3389/fpsyg.2022.968684

**Published:** 2022-09-30

**Authors:** Chien Chou Yu, Jin Hua Luo

**Affiliations:** School of Economics and Management, Sanming University, Sanming, China

**Keywords:** project procurement management process, negotiation process, win-win project procurement negotiations, fuzzy set theory, fuzzy TOPSIS

## Abstract

In recent years, organizations worldwide have widely applied the project approach in business and value delivery. Negotiation is essential to the success of a project; however, it has not been explored systematically in the project context. A gap remains between knowledge and practical behavior during negotiation settlements throughout projects. Many project procurement (PP) negotiations do not work as expected. This study develops a practical framework using the scientific method to help close the gap and improve PP negotiations. The proposed framework uses the fuzzy TOPSIS (technique for order preference by similarity to ideal solution) method to integrate the PP management process (PPMP) and the three-phase negotiating model. Through this approach, notable variables and potential solutions under uncertain negotiation situations are quantitatively examined in the early stage and managed until the completion of PP. Thus, expected agreements can be obtained in a timely and efficient manner, with negotiating parties committing to implementing what has been agreed on. Such a commitment facilitates win-win outcomes. An example is presented to demonstrate how the proposed framework operates, and practical implications for managers of project-based organizations are offered. This study provides researchers and practitioners with a foundation to study refined models to enhance project negotiations with interdisciplinary integration.

## Introduction

In recent years, organizations worldwide have widely applied the project approach in business activities and value delivery to stakeholders. A project can be defined as a temporary effort to produce unique goods, services, and outcomes to achieve the predetermined goals of an organization or society (Project Management Institute, 2018). Currently, the type and scope of projects in an organization include product development, construction, infrastructure maintenance, information system installation and update, business consulting and training services, etc. Most projects are completed by cross-organizational partners within the constraints of time, cost, scope, manpower, quality, and risk. A project’s success has direct and indirect impacts on organizational effectiveness, socioeconomic development, and sustainability (Trocki et al., 2020; Ladika and Budiman, 2022).

A successful project is mainly characterized as delivering qualified and customer-satisfied results within given resources. To improve the success rate of the project, the project management adopts the life cycle model, which divides the project work into different stages, and then utilize sound management approaches to reduce the risk and uncertainty of the work over the project lifecycle (Project Management Institute, 2021; Guğmundsdóttir et al., 2022). Additionally, a project is done by people. To ensure the success of the project and project management, the personnel involved in the project should have the complete competences to grasp the environmental factors, make good use of the best practices, and create a teamwork environment to complete the project work, obtain the optimal results, and create value for the organization and society (Zuo et al., 2018; Shaukat et al., 2022).

Negotiations, a key business strategy, managerial competence, and operational tool, are essential to a project’s success (IPMA and ICB-OCB-IPMA, 2015; Project Management Institute, 2018). Project managers must negotiate the allocation of resources from top authorities and discuss concerns regarding staffing and technical allocation with functional managers. They also frequently communicate with management teams for projects, programs, and portfolios with reference to priorities and conflicts. Successful negotiations enable project parties to resolve differences, reach favorable agreements, maintain collaborative relationships, and work toward win–win business outcomes (Larson and Gray, 2021). The successful negotiation is especially relevant for project procurement (PP) in an organization.

In the ever-changing business environment, projects are rarely undertaken solely within organizations. Project team members must purchase goods and services from external sources to accomplish organizational objectives (Project Management Institute, 2021) such as developing new investment systems, bespoke products, and specialized consultant services. PP involves implicit and explicit concerns regarding conflicting interests between buyers and sellers, resulting in complex negotiations involving complexity, uncertainty, and dynamicity (Baily et al., 2015). Given the diversity and uncertainty of PPs, most negotiations involve challenges associated with relying on automated market mechanisms. Face-to-face negotiations begin in the early stage of PPs but do not necessarily end when an agreement is reached. Disputes or new concerns typically arise during the implementation of the agreement. Theoretically, Negotiations for PPs are a two-part process. In the first part, an agreement is reached. The second part involves implementation of that agreement. The outcome of implementation and the process through which that agreement is reached determine the success of a PP negotiation (Larson and Gray, 2021).

Although negotiation is the preferred method to solve PP problems in terms of time, cost, and outcomes, practical surveys of hundreds of negotiated projects have revealed that despite the comprehensive and creative crafting of agreement terms during negotiations, most agreements are affected by future trends, and further improvement is required during the agreement implementation process (Turner and Keegan, 2001; Ertel, 2004; Kujala et al., 2007; Liu and Cheah, 2009; Yang et al., 2010). Studies on project negotiations involving decision analysis have not explored negotiations systematically in the context of projects. Most decision analysis tools developed for negotiations are used to reach favorable agreements (Spector, 1993; Sebenius, 2009; Wang et al., 2011). A gap remains between knowledge and practical behavior during negotiation settlements throughout projects (Murtoaro and Kujala, 2007). Although certain models based on decision-making theories have been developed to facilitate negotiations, business parties may conceal information to safeguard potential benefits (Yang et al., 2010). In addition, the information required for negotiation models to analyze decisions is often insufficient, unavailable, or costly to obtain (Baily et al., 2015). Fuzzy measurement can be applied to manage scenarios in which information is insufficient or unavailable and to increase the accuracy business negotiation analyses (Xu and Chen, 2007).

From the behavioral perspective, Fisher and Ury (1983) repeatedly emphasized that negotiation is not a game of winning against another party. Win–win collaboration enables mutually satisfactory solutions and favorable relationships between parties, which are key for parties to achieving expected tasks during solution implementation. Various principles for cooperative negotiation have been proposed, including preparing before action, separating people from problems, focusing on interests rather than positions, presenting a range of options, building long-term partnerships, and maintaining resilience (Fisher et al., 2011). Raiffa (1985) proposed postsettlement settlement, noting that most settlement negotiators leave room for settlements that are favorable to both parties because of a lack of information. Susskind (2017) identified collaborative relationships and willingness to share information as two critical elements that facilitate postsettlement settlement in negotiations. From a macro standpoint, Baber (2018) indicated that negotiated agreements should be comprehensively monitored and evaluated during their implementation. Smolinski and Xiong (2020) asserted that the postsettlement settlement is crucial for resolving conflicts in the increasingly complex modern business environment. Yu et al. (2021) proposed a hybrid multiple attribute decision-making (MADM) approach for ensuring negotiated agreements is practical and operational in procurement negotiations. However, this approach does not involve the use of the fuzzy technique to examine procurement negotiations when the amount of information is insufficient.

The above findings suggest that having an appropriate fuzzy decision model that enables project participants to execute and manage complex negotiations in a timely and efficient manner throughout the PP process is critical to project success. Considering the theoretical and practical gaps in the project area, many PP negotiations do not proceed as agreed; this study aims to develop a practical framework using scientific methods to help bridge the gap and better manage PP negotiations. This study integrates the PP Management Process (PPMP) and the Three-Phase Negotiation Model (TPNM) into a systematic framework using the fuzzy TOPSIS (through sequential preference technique similar to ideal solutions) method to quantitatively examine negotiation variables and potential solutions to facilitate management until the PP is completed. More specifically, the proposed framework uses two sets of data (initial offers from negotiators) to build a space of possible negotiated agreements (SPAN) in which all possible alternatives available to negotiators can be comprehensively evaluated and prioritized before and during bargaining; this enables a timely and efficient agreement at the negotiating table with adequate information. The framework also enhances the negotiation process by adding a reconciliation step after an agreement is reached. During this step, the negotiating parties can make final adjustments to the agreement by exchanging previously undisclosed information. This step increases the negotiating parties’ satisfaction with the negotiation, thereby strengthening their commitment to a win-win outcome.

A numerical example demonstrates how the proposed model operates. Research has demonstrated that negotiations for PP operate well under the proposed framework. Given that the structure and processes of negotiations are the same across different areas of business, with minor adjustments, the proposed framework can be generalized to other project negotiation settings, such as those involving resources, stakeholders, and risk allocation, to facilitate agreement, resolve disputes, maintain relationships, and ensure sustainable win-win project outcomes. This study provides a foundation for study of refined models to enhance project negotiations with interdisciplinary integration. The remainder of this paper is organized as follows. Section “Literature review” reviews the literature on which the proposed model is based. Section “The fuzzy TOPSIS framework for win-win PP negotiations” presents basic definitions of fuzzy set theory and fuzzy TOPSIS. Section “Proposed PP negotiation framework” introduces the proposed model. Section “Numerical example” applies a numerical example to demonstrate how the proposed model operates. Section “Conclusion” summarizes the findings and concludes the paper.

## Literature review

This section briefly introduces the PPMP, the TPNM, and key variables that affect the process and outcomes of the negotiation process. The literature review serves as the basis for the proposed framework.

### Project procurement management process

A project can be defined as a complex, nonroutine, one-time effort to satisfy customers limited by time, budget, resources, and performance specifications (Larson and Gray, 2021). PP involves acquiring the goods, services, or outcomes required to achieve projects’ objectives from outside the responsible organization (Baily et al., 2015). In certain situations, multiple processes might be required to support complex procurement; in others, a simple process might be adequate for managing a re-buy commodity. This paper introduces the PPMP, which was developed by the Project Management Institute and is commonly recognized by project practitioners worldwide as a standard framework for handling complex PPs (Project Management Institute, 2021).

The PPMP consists of four subprocesses: planning procurement, conducting procurement, administering procurement, and closing procurement (Project Management Institute, 2018). In these subprocesses, the project manager must first ensure that procurement is the optimal means of achieving the project objectives, in terms of scope, schedule, resources, quality, constraints, and associated risks. Next, the procurement statement of work is developed, qualified sellers willing to provide required items are identified, resources to be expended are estimated, the type of contract required to minimize procurement risk is determined, and source selection criteria are designated. Procurement documentation in the form of a request for proposal or quotation is then produced for solicitation. On the receipt of sellers’ responses, a committee applies previously determined source selection criteria to the selection of qualified sellers for negotiation (Larson and Gray, 2021).

The main purpose of negotiations is to determine the terms of purchase between the buyer and supplier and to close a contract in accordance with the terms of agreement (Smolinski and Xiong, 2020). The terms to negotiate and finalize in a contract typically include the price, payment, quality, packaging, transportation, insurance, delivery, inspection, acceptance, warranty, and after-sales services. They may also involve administrative measures and legal protections related to contract performance (Baily et al., 2015). However, given the inherent uncertainty in most project work, no contract can address all possible terms. Negotiations can occur at any point during the PPMP, and the negotiation process is a subordinate process of PP for resolving differences of opinion between buyers and sellers during contract transactions (Larson and Gray, 2021).

### Three-Phase Negotiation Model

Each negotiation is unique to each project, but the structure of negotiations is generally the same (Kujala et al., 2007). Numerous procurement researchers and practitioners around the world have adopted TPNM, a conceptual model introduced by Baily et al. (2015), as a foundation for developing negotiation skills. The TPNM divides the negotiation process into three stages: prenegotiation, meeting, and post-negotiation. The prenegotiation stage involves preparation for negotiation, gathering information, identifying challenges, setting objectives, analyzing potential agreements, and developing strategies and tactics. The meeting stage involves introductions, clarification, discussions, concessions, deadlock breaking, and agreement. The post-negotiation stage involves the implementation of the agreements reached during the meeting stage. The success of the TPNM depends on control and management of variables that affect the process and outcomes of the TPNM.

Information is the key to successful negotiations (Atkin and Rinehart, 2006). PP negotiations often require the collection, aggregation, and analysis of large amounts of information early in the negotiation process, and the type of information varies by negotiation. The ability to obtain the information required for negotiations is subject to temporal and resource constraints. Obtaining an adequate amount of information is challenging (Lewicki and Hiam, 2011), and inadequate amounts can strongly affect the process.

Issues related to PP negotiations often arise from disagreements regarding proposals between buyers and sellers (Sebenius, 2009), which occur when buyers and sellers make different claims based on the procurement documents. Disagreements are attributable to differences in views regarding objectives, price, specifications, delivery, inspection, financing, law, labor, and after-sales arrangements between parties (Baldi et al., 2016). Each issue requires a different space for discussion and carries a different weight for each party, depending on a combination of their perceptions and actual interests on the issue. Understanding each party’s concerns and priorities is a vital to success (Yu et al., 2021).

Objectives result from negotiators’ assessments of the conditions under which each problem can be negotiated (Baily et al., 2015). Such an assessment typically yields a range of options. The upper end of the range represents the least desirable outcome (LDO) or a bottom line representing a point that negotiators will not exceed. The lower end of the range can be the most desirable outcome (MDO) or an opening price representing the first bid of a negotiator. In a PP negotiation, each party knows the MDO of the other party because of quotations and proposals. However, one side can only infer the LDO of the other side. LDOs and MDOs on both sides usually go in opposite directions, with the LDO being the high price for the buyer and the low price for the seller. Thus, the distance between two LDOs constitutes a zone of possible agreement (ZOPA; Fisher et al., 2011), within which a range of mutually possible satisfactory outcomes can be analyzed. However, a ZOPA may not exist. In such a situation, a gap between buyers and sellers ensues. To close a deal, one or both parties must adjust their range until a ZOPA emerges. Otherwise, negotiation may not be the appropriate approach, and alternatives should be considered.

**FIGURE 1:**
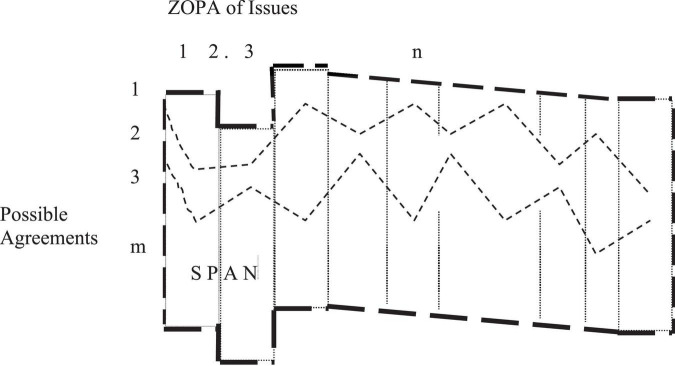
Graphical representation of the SPAN.

Negotiators typically resolve problems involving gaps between buyers and sellers before a ZOPA emerges and agreements between alternatives within that ZOPA are reached. However, in most negotiations, multiple issues may need to be agreed on. A negotiated package of issues is called a negotiating mix (Fisher and Ury, 1983). Each issue in the mix has its own ZOPA. By considering all ZOPAs together, a SPAN can be established. The SPAN can be established by two sets of LDOs or MDOs for all issues. In a SPAN, all possible agreements can be formulated and analyzed by connecting the various points of each issue in ZOPAs. A graphical representation of a SPAN is presented in Figure 1.

For *n* issues to negotiate, each issue has its own ZOPA, represented by a vertical rectangle covered by a small dotted line (Figure 1). The SPAN is displayed as an area covered by a large bold dotted line. Of the *m* possible agreements within the SPAN, two are presented as small dotted lines horizontally linking issues 1 to *n*. Negotiators can use possible agreements to determine negotiation objectives and to prepare strategies, tactics, and concessions for attaining aspired outcomes.

Strategies are parties’ plans, directions, or positions during negotiations (Mintzberg and Quinn, 1991). According to Lewicki and Hiam (2011), win–lose (competitive or allocated) and win–win (collaborative or integrated) negotiation strategies have received considerable attention from scholars. In a win–lose negotiation situation, resources are theoretically limited, and the objective of a party is usually to maximize its share of the resources. By contrast, in a win–win negotiation situation, the objectives of the parties are not mutually exclusive; the gain of one party is not necessarily at the expense of the other party.

Generally, competitive negotiation is easy to obtain relatively short-term gains, but it is expensive and time-consuming and can easily lead to a deadlock or failure of the negotiation. In addition, both negotiation parties will conceal information and mislead the other party for their gain, thereby damaging the long-term relationship between two parties. Worse, the final agreement could become a later dispute. Typically, competitive negotiation takes place in a procurement setting, where the procurement is a one-time deal, and the outcomes and future relationships of the negotiation are not important. Conversely, cooperative negotiation is a problem-solving-oriented interactive approach in which both parties express and understand each other’s positions and expectations and seek win-win results that may create the highest value for both parties (Lewicki and Hiam, 2011).

In practice, the above two negotiation strategies are mixed in PP. Negotiators need to look at the actual situation and adopt strategies accordingly. Especially in cross-cultural negotiation, negotiators need to flexibly adopt appropriate negotiation strategies according to the customs, beliefs, and laws of different regions and countries. For example, one may focus on the long-term relationship and the other on the agreements. In one country, reaching an agreement is significant while in another country, the agreement implementation is the focal point (Saee, 2008). According to Gulbro and Herbig (1995), Americans are accustomed to using competitive negotiation based on interests, while Chinese and Japanese prefer relationship-based negotiation.

In the PP context, a win–win negotiation is preferred because it promotes innovation, reasonable decision-making, and creative problem-solving (Project Management Institute, 2021). However, a win–lose scenario may initially emerge in negotiations where the parties seek to maximize their own shares of resources. Through serious discussion and mutual exploration, negotiation can lead to win–win options (Olekalns et al., 2003). To defend against irrational or win–lose negotiators, developing a robust best alternative to a negotiated agreement (BATNA) is imperative. A strong BATNA equates to a strong negotiating position, confident negotiating team, and clear deadline for reaching a negotiated agreement. With a BATNA, negotiators are empowered and know when to say “no deal” if all parties do not agree to work toward a win–win situation (Larson and Gray, 2021).

Tactics are adaptive moves designed to ensure short-term gain. Studies on negotiation tactics have identified the following approaches: take-it-or-leave-it, bogey, crunch, auction, and good guys–bad guys. However, experienced negotiators have noted that although the appropriate use of tactics may lead to successful negotiations, relying solely on tactics tends to damage long-term relationships and should be avoided (Karass, 1974). Therefore, knowing how to use suitable tactics at different stages of the negotiation process how opponents use them is critical to a successful negotiation (Baily et al., 2015).

Concessions are prearranged giveaway items in the negotiating objectives offered by one party to the other to encourage reciprocation. Negotiations cannot proceed without concessions (Lewicki et al., 1994). At the beginning of a negotiation, negotiators usually present their opening offers and then reach a compromise. The flexibility between the opening offers of the parties and what they are willing to accept enables concessions. Negotiators can determine whether to make concessions but asking for more than a previous request is not an option. Concession negotiation proceeds under two basic modes: large concessions and small concessions. Giving large concessions can win goodwill, but it may also make the other party feel that their offer is of little value. In addition, it may raise the expectations and demands of the other party regarding concessions. Small concessions may be rewarded more by the other party, but they may also cause one or both parties to become uncompromising (Raiffa, 2007; Lewicki and Hiam, 2011). Skilled negotiators often prepare potential solutions with a package approach that they but not the other party consider holding approximately the same value; for this approach generally increases negotiation flexibility and leads to satisfactory negotiation outcomes (Fisher et al., 2011).

As discussed above, all variables in the negotiation process are dynamic, involving multiple options that affect the process or outcomes. Currently, several multiple criteria decision-making (MCDM) methods have been proposed to help negotiating parties examine negotiating issues, evaluate alternatives that are satisfactory to both parties, and resolve differences (Murtoaro and Kujala, 2007). MCDM models contains two main categories; the multi-objective decision model (MODM) for planning and design of problems; the multi-attribute decision model (MADM) for ranking and evaluation of problems (Liou and Tzeng, 2012). The main purpose of this research is to establish an applicable negotiation framework to improve the gap between theory and practice in the current PP negotiation setting. The framework to be proposed requires ranking and evaluating negotiation issues and alternatives in making quick decisions under uncertainty and dynamic situations. Furthermore, the framework must be easy to use in practice. Therefore, The Fuzzy TOPSIS method within the MADM family is adopted as the methodology to design the proposed framework and introduce in the next section.

## The fuzzy TOPSIS framework for win-win PP negotiations

This section first briefly introduces the essential concepts and computational equations related to the Fuzzy set theory, Fuzzy TOPSIS, and the proposed framework.

### Fuzzy set theory

*U* = {*u*_1_,*u*_2_,⋯,*u*_*n*_} represents the universe of discourse. Fuzzy set $\tilde{A}$ in *U* is represented by $\{\left(u,f_{\tilde{A}}\left(u\right)|u\in U\right\}$, where $f_{\tilde{A}}$ is the membership function of $\tilde{A}$, $f_{\tilde{A}}$: *U*→ [0, 1] and $f_{\tilde{A}}\,\left(u\right)$ represents the grade of membership of $\tilde{A}$ (Zadeh, 1965). A fuzzy number is a fuzzy subset in *U*, which is both convex and normal. A fuzzy number can be of any shape. The triangular form of the membership function is a simple and effective means of formulating decision-making problems and is used in relevant calculations (Xu and Chen, 2007). This study uses triangular fuzzy number $\tilde{A}$ defined by triplet (*a*_1_,*a*_2_,*a*_3_) with a membership function as follows:


(1) $$f_{\tilde{A}}\,\left(u\right)=\left\{ \begin{array}{cccc} 0, & & u<a_{1} & \\ \frac{u-a_{1}}{a_{2}-a_{1}}, & & a_{1}\leq u\leq a_{2} & \\ \frac{a_{3}-u}{a_{3}-a_{2}}, & & a_{2}<u\leq a_{3} & \\ 0, & & a_{3}<u & \end{array}\right.$$

$\tilde{A}=\left(a_{1},a_{2},a_{3}\right)$ and $\tilde{B}=\left(b_{1},b_{2},b_{3}\right)$ are two triangular fuzzy numbers. The arithmetic for $\tilde{A}$ and $\tilde{B}$ is as follows (Chiou et al., 2005):


(2) $$\tilde{A}\,\left(+\right)\,\tilde{B}=\left(a_{1}+b_{1},a_{2}+b_{2},a_{3}+b_{3}\right)$$

(3) $$\tilde{A}\,\left(-\right)\,\tilde{B}=\left(a_{1}-b_{3},a_{2}-b_{2},a_{3}-b_{1}\right)$$

(4) $$\tilde{A}\,\left(\times \right)\,\tilde{B}=\left(a_{1}\,b_{1},a_{2}\,b_{2},a_{3}\,b_{3}\right)$$

(5) $$\tilde{A}\,\left(/\right)\,\tilde{B}=\left(a_{1}/b_{3},a_{2}/b_{2},a_{3}/b_{1}\right)$$

(6) $$k\,\tilde{A}=\left(k\,a_{1},k\,a_{2},k\,a_{3}\right)$$

(7) $$D\,\left(\tilde{A},\tilde{B}\right)=\sqrt{\frac{1}{3}\,\left[{\left(a_{1}-b_{1}\right)}^{2}+{\left(a_{2}-b_{2}\right)}^{2}+{\left(a_{3}-b_{3}\right)}^{2}\right]},$$

where *D* is the distance between $\tilde{A}$ and $\tilde{B}$.

Linguistic variables (Zadeh, 1975) are variables with values presented in linguistic terms. In MADM, the weights of various attributes and the rating of qualitative alternatives are regarded as linguistic variables, the values of which are taken from linguistic terms and can be represented by triangular fuzzy numbers (Tables 1, 2; Chen, 2000). $\tilde{M}$ is a fuzzy matrix if at least an entry in $\tilde{M}$ is a fuzzy number (Buckley, 1985).

**TABLE 1:** Fuzzy linguistic variables for weights of various attributes.

Linguistic terms	Abbreviation	Fuzzy numbers
Very Low	VL	(0.00, 0.00, 0.20)
Low	L	(0.05, 0.20, 0.35)
Medium Low	ML	(0.20, 0.35, 0.50)
Medium	M	(0.35, 0.50, 0.65)
Medium High	MH	(0.50, 0.65, 0.80)
High	H	(0.65, 0.80, 0.95)
Very High	VH	(0.80, 1.00, 1.00)

**TABLE 2:** Fuzzy linguistic variables for qualitative attribute rating.

Linguistic terms	Abbreviation	Fuzzy numbers
Very Poor	VP	(0, 0, 1)
Poor	P	(0, 1, 3)
Medium Poor	MP	(1, 3, 5)
Fair	F	(3, 5, 7)
Medium Good	MG	(5, 7, 9)
Good	G	(7, 9, 10)
Very Good	VG	(9, 10, 10)

### Fuzzy TOPSIS

The TOPSIS approach, introduced by Yoon and Hwang (1995), has been widely applied to various types of MADM problems (Chen, 2000; Chiou et al., 2005; Shamsuzzoha et al., 2021). In TOPSIS, the most preferred alternative is assumed to have the shortest distance from the positive ideal solution (PIS) and the longest distance from the negative ideal solution (NIS). An ideal solution is defined as a set of ideal values for all attributes considered. The PIS and NIS are a collection of the most and least attainable values for all attributes, respectively. Based on PIS and NIS, an index of similarity to the PIS is calculated in TOPSIS by combining the proximity to the PIS with the remoteness from the NIS. The order of preference of all alternatives and a most acceptable alternative can then be determined accordingly (Nãdãban et al., 2016).

Critics of TOPSIS, however, have indicated that crisp numbers used to weigh attributes or to rate alternatives cannot adequately model real-life decision-making problems (Chen, 2000). Perfect knowledge is not always available, and complete information is not easily obtained. With the development of fuzzy set theory (Zadeh, 1965), fuzzy TOPSIS can be employed such that these shortcomings can be overcome to a large extent. Fuzzy TOPSIS uses linguistic terms to represent the weight of attributes and the rating of alternatives, thus enabling decision-makers to incorporate unquantifiable, incomplete, and unobtainable information into the decision-making model (Kutlu and Kahraman, 2019).

Currently, classical fuzzy TOPSIS using fuzzy set was applied most widely and frequently, however, its variants using intuitionistic, neutrosophic set, single-value neutrosophic set, hesitant or 2-type fuzzy sets, among others are also available for use in more complex scenarios (Biswas et al., 2016; Palczewski and Sałabun, 2019). This study adopts classical fuzzy TOPSIS to ensure the proposed framework to be operable and applicable. According to Shamsuzzoha et al. (2021), the classical fuzzy TOPSIS process comprises the following steps:

Step 1: construction of the fuzzy decision matrix.


(8) $$\begin{array}{c} \begin{array}{cccccccc} & & C_{1} & C_{2} & \,\cdots & C\,n & & \end{array}\\ \tilde{M}\;=\begin{array}{c} A_{1}\\ A_{2}\\ \vdots \\ A_{m} \end{array}\left[\begin{array}{cccc} {\tilde{x}}_{11} & {\tilde{x}}_{12} & \cdots & {\tilde{x}}_{1\,n}\\ {\tilde{x}}_{21} & {\tilde{x}}_{22} & \cdots & {\tilde{x}}_{2\,n}\\ \cdots & \cdots & \cdots & \cdots \\ {\tilde{x}}_{m\,1} & {\tilde{x}}_{m\,2} & \cdots & {\tilde{x}}_{m\,n} \end{array}\right] \end{array}$$

(9) $$\tilde{W}=\text{[}{\tilde{w}}_{1},{\tilde{w}}_{2},\cdots ,{\tilde{w}}_{n}]$$

where ${\tilde{x}}_{i\,j}$ is the rating of *i*th alternative *A*_*i*_ with respect to *j*th attribute *C*_*j*_; ${\tilde{w}}_{j}$ is the weight of *j*th attribute *C*_*j*_; and ${\tilde{x}}_{i\,j},{\tilde{w}}_{j},i=1,2,\cdots ,m;j=1,2,\cdots ,n$ are linguistic variables described by triangular fuzzy numbers ${\tilde{x}}_{i\,j}=\left(a_{i\,j\,1},a_{i\,j\,2},a_{i\,j\,3}\right),{\tilde{w}}_{j}=\left(w_{j\,1},w_{j\,2},w_{j\,3}\right).$


(10) $${\tilde{x}}_{i\,j}=\frac{1}{k}\text{[}{\tilde{x}}_{i\,j}^{1}+{\tilde{x}}_{i\,j}^{2}+\cdots +{\tilde{x}}_{i\,j}^{k}]$$

(11) $${\tilde{w}}_{j}=\frac{1}{k}\text{[}{\tilde{w}}_{j}^{1}\left(+\right){\tilde{w}}_{j}^{2}\left(+\right)\cdots \left(+\right){\tilde{w}}_{j}^{k}]$$

${\tilde{x}}_{i\,j}^{k}\,\text{and}\,{\tilde{w}}_{i\,j}^{k}$ are the rating and weight of the *k*th decision-maker, respectively.

Step 2: construction of the normalized fuzzy decision matrix.


(12) $$\tilde{R}=\text{[}{\tilde{r}}_{i\,j}]{}_{m\times n}$$

where $r_{i\,j}=\left(\frac{a_{i\,j\,1}}{a_{j}^{*}},\frac{a_{i\,j\,2}}{a_{j}^{*}},\frac{a_{i\,j\,3}}{a_{j}^{*}}\right),j\in J_{1},a_{j}^{*}=\underset{1\leq i\leq m}{m\,a\,x}a_{i\,j\,3};$$r_{i\,j}=\left(\frac{a_{j}^{*}}{a_{i\,j\,3}},\frac{a_{j}^{*}}{a_{i\,j\,2}},\frac{a_{j}^{*}}{a_{i\,j\,1}}\right),j\in J_{2};\text{and}\,a_{j}^{*}=\underset{1\leq i\leq m}{m\,i\,n}a_{i\,j\,1}$. *J*_1_ is a set of benefit attributes, and *J*_*2*_ is a set of cost attributes.

Step 3: construction of the weighted normalized fuzzy decision matrix.


(13) $$\tilde{V}=\text{[}{\tilde{v}}_{i\,j}]{}_{m\times n},i=1,2,\cdots ,m,j=1,2,\cdots ,n,$$

where ${\tilde{v}}_{i\,j}={\tilde{r}}_{i\,j}\,\left(\times \right)\,{\tilde{w}}_{j}$.

Step 4: identification of fuzzy positive ideal solution (*FPIS*) and fuzzy negative ideal solution (*FNIS*).


(14) F⁢P⁢I⁢S={v~1+,v~2+,⋯,v~n+}={m⁢a⁢x 1≤i≤mv~i⁢j}

(15) F⁢N⁢I⁢S={v~1-,v~2-,⋯,v~n-}={m⁢i⁢n 1≤i≤mv~i⁢j}

where *j* = 1, 2, …, *n*.

Step 5: calculation of the separation measures of each alternative from the *FPIS* and the *FNIS*:


(16) $$S_{i}^{+}=\sqrt{\sum_{j=1}^{n}\text{[}D\left({\tilde{v}}_{i\,j},{\tilde{v}}_{j}^{+}\right)]{}^{2}}$$

(17) $$S_{i}^{-}=\sqrt{\sum_{j=1}^{n}\text{[}D\left({\tilde{v}}_{i\,j},{\tilde{v}}_{j}^{-}\right)]{}^{2}}$$

where⁢i=1,2,⋯,m.

Step 6: calculation of the closeness coefficient.


(18) $$C\,C_{i}^{*}=\frac{S_{i}^{-}}{\left(S_{i}^{+}+S_{i}^{-}\right)}\,i=1,2,\cdots ,m;$$

Step 7: ranking of preferences in descending order of $C\,C_{i}^{*}$. The order of preference for all alternatives is determined, and the one with the maximum $C\,C_{i}^{*}$ is the optimal alternative.

### Proposed PP negotiation framework

**FIGURE 2:**
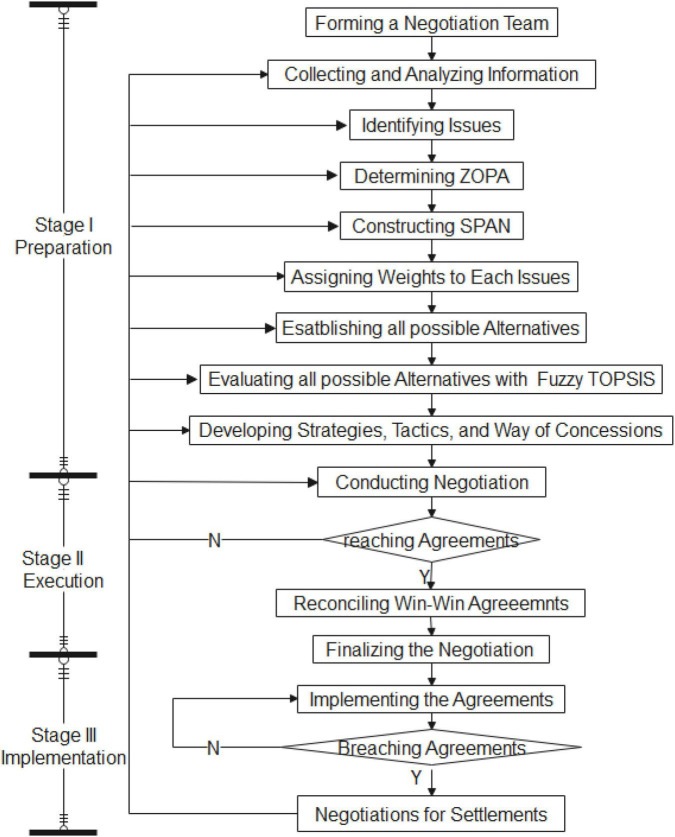
An integrated model for negotiation in project procurement.

Considering all issues as attributes and all possible agreements in a SPAN as alternatives (Please refer to Figure 1), the proposed framework formulates PP negotiation problem into a MADM format, then use fuzzy TOPSIS to evaluate all criteria and alternatives as the basis for preparing and executing negotiation strategies, tactics, and ways of concessions with sufficient information before and during negotiations, and thus obtain expected results. The proposed framework contains three main stages, namely preparation, execution, and implementation. A graphical representation of the proposed framework is shown in Figure 2. The proposed framework is based on the perspective of a buyer with a win–win negotiating intent in a PPMP setting. The framework procedure is described as follows.

#### Stage 1: Preparation

First, a negotiation team (NT) is formed with team members from appropriate disciplines. After collecting and analyzing information, the NT identifies all issues to negotiate (Eq. 19) along with the respective ZOPAs (Eq. 20):


(19) $$IS_{j},j=1,2,\cdots ,n.$$

(20) $$Z\,O\,P\,A_{j}=\begin{array}{cc} \left[I\,S_{j}^{+},I\,S_{j}^{-}\right], & j=1,2,\cdots ,n. \end{array}$$

where *n* is the number of issues and ZOPAs identified by the NT, and $I\,S_{j}^{+}$ and $I\,S_{j}^{-}$ are the upper and lower limits of each *ZOPA*_*j*_, respectively.

As indicated in Table 1 and Eq. 11, the NT members assign a weight to each issue based on its importance. The weights are expressed as


(21) $$\tilde{W}=\left[{\tilde{w}}_{j}\right],j=1,2,\cdots ,n.$$

Based on Eqs 19, 20, a SPAN is identified as follows:


(22) $$S\,P\,A\,N={\left[S\,P_{i\,j}\right]}_{2\times n},i=1,2;j=1,2,\cdots ,n,$$

where [*SP*_1j_] and [*SP*_2j_] are a collection of $I\,S_{j}^{+}$ and $I\,S_{j}^{-}$from all *ZOPA*_*j*_, respectively.

Based on Eq. 22, the NT considers a set of alternatives between the [*SP*_1j_] and [*SP*_2j_] as possible agreements as follows:


(23) $$P\,A={\left[A_{i\,j}\right]}_{m\times n},\begin{array}{cc} i=1,2,\cdots ,m; & j=1,2,\cdots ,n. \end{array}$$

where *m* is the number of alternatives and *n* is the number of negotiation issues.

Based on Eqs 21, 23, a decision matrix is formed in which all alternatives (possible agreements) considered by the NT can be evaluated and prioritized through the fuzzy TOPSIS process. However, the negotiation issues (attributes) may be quantitative, such as price, or qualitative, such as applicable laws, such that the *A_ij_* in the PA (Eq. 23)can be either a numerical or linguistic term. To evaluate all alternatives (possible agreements) in the PA, *A*_*ij*_ is normalized through several methods.

i) Quantitative attributes:

Quantitative attributes are normalized into interval [0, 1] by using the linear scale method.


(24) $$r_{i\,j}=\frac{A_{i\,j}}{\underset{1\leq i\leq m}{m\,a\,x}A_{i\,j}},j\in J_{1}$$

(25) $$r_{i\,j}=\frac{\underset{1\leq i\leq m}{m\,i\,n}A_{i\,j}}{A_{i\,j}},j\in J_{2}$$

*J*_1_*andJ*_2_ are sets of benefit and cost attributes, respectively.

ii) Qualitative attributes

The NT first assigns linguistic values to the linguistic terms: ${\tilde{A}}_{i\,j}\,\left(a_{i\,j\,1},a_{i\,j\,2},a_{i\,j\,3}\right)$ (Table 2). Next, by using Eq. 12 in step 2 of Section “Fuzzy TOPSIS,” each ${\tilde{A}}_{i\,j}$ is transformed into a normalized value denoted by ${\tilde{r}}_{i\,j}$.

By following steps 3–7 in Section “Fuzzy TOPSIS,” the $C\,C_{i}^{*}$ of all possible agreements in the PA can be computed and arranged in descending order.


(26) $$C\,C=\left\{C\,C^{1},C\,C^{2},\cdots ,C\,C^{1},\cdots ,C\,C^{m-1},C\,C^{m}\right\}$$

**FIGURE 3:**
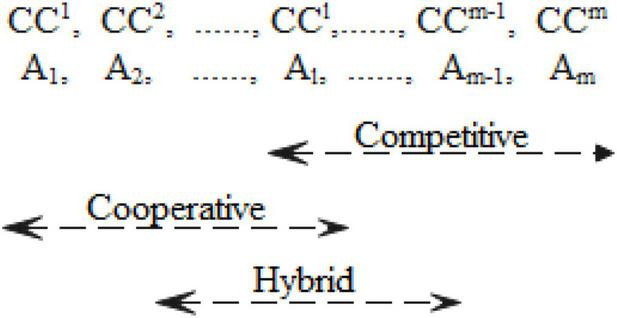
Determination of negotiating strategies.

Based on Eq. 26, the NT can consider objectives and strategies as follows:


(27) $$S\,T^{v}=\left[C\,C^{L},C\,C^{U}\right],$$

where *v* is the number of strategies considered by the *NT* and [*CC*_*L*_,*CC*^*U*^] is a range in which *CC*^*L*^ and *CC*^*U*^ are the smallest and the largest values among all CC values within the corresponding strategy, respectively. A wider range of *ST*^*v*^ covering small CC values implies a win–win strategy. Conversely, a narrower range of *ST*^*v*^ covering large CC values implies a win–lose strategy. A range that lies in the middle of the CC values implies that *ST*^*v*^ is a hybrid strategy. A graphical representation for determining negotiating strategies is displayed in Figure 3.

Based on Eqs 26, 27, the NT can develop appropriate tactics, concession methods, and a BATNA based on the quantified CC values.

Finally, the information concerning the ideal solution, ZOPAs, SPAN, strategies, objectives, tactics, concession methods, and BATNA generated from the preceding steps is incorporated into a negotiation plan to guide the negotiation process.

#### Stage 2: Execution

In this stage, the negotiating parties meet to discuss their differences, break deadlocks, and reach an agreement based on their respective negotiation plans. Once an agreement is reached, the negotiating parties disclose all information used to develop their negotiation plans. Based on this new information, the two parties work in concert under the proposed framework to jointly determine whether preferable alternatives exist.

Notably, a more satisfactory agreement may be reached by adjusting the weights previously assigned to each issue. However, this cannot involve changing the negotiated issues. A more favorable outcome depends on the order in which the parties rank the alternatives. Finally, based on the agreement reached, the negotiating parties sign a contract to execute specific tasks related to the PP.

#### Stage 3: Implementation

This stage involves the implementation of the contract signed in the previous stage. The seller is typically mainly responsible for the production and delivery of contract items, whereas the buyer is mainly responsible for inspection and payment. Negotiations may be the preferred option for solving disputes when either party defaults or raises new concerns. The negotiation variable data analyzed through the fuzzy TOPSIS framework in the preparation stage can provide valuable information and help contracting parties establish a basis for collaboration such that win–win outcomes can be attained. For example, the separation measures and weights on issues and closeness coefficients regarding the possible agreements can serve as specific references for both parties to understand potential interests rather than positions as well as the options considered by both parties. Thus, new issues can be considered; moreover, a common basis for a mutually satisfactory solution can be reached.

## Numerical example

Suppose company B is pursuing a contract with company S to procure the equipment for a project within a limited time. This PP is governed by the PPMP. The current process has passed the planning phase. However, companies B and S disagree on several issues regarding purchasing. These differences are not resolved through discussion, and the two sides decide to arrange a negotiation meeting for settlement. The proposed framework is as follows. All calculations in the numerical case are done using MS Excel 2017.

### Preparation stage

Company B forms an NT with five members, namely *NT*_1_,*NT*_2_,*NT*_3_,*NT*_4_,*andNT*_5_,to analyze pertinent information and to identify negotiation issues and their corresponding ZOPAs. Four issues and their corresponding ZOPAs are presented in Table 3.

**TABLE 3:** Negotiation issues and correspondent *ZOPA*s.

Issues	*ZOPA*s	Note
Price of the equipment (PE)	[16, 20]	The S quoted 20 million US dollars for selling of the equipment, but the B only wanted to pay for 16 million US dollars.
Delivery schedule (DS)	[8, 12]	The B requested that all contract items be delivered within 8 months after the effectiveness of the contract, but the S can only commit to deliver within 12 months.
Warranty period (WP)	[2, 1]	The B requested the S provide a 2-year warranty on deliverables of contract, but the S counter-offered 1 year warranty.
Applicable law (AL)	[DC, LA]	The B wishes to use DC law to govern the contract, but the S prefers LA law.

In Table 3, the price of the equipment and the delivery schedule are quantitative cost attributes, and the warranty period is a quantitative benefit attribute. Applicable laws constitute a qualitative attribute in linguistic terms.

The NT establishes a SPAN (Table 4) from which 13 alternatives (Table 5) are selected as possible agreements for use in settling disagreements in the execution stage.

**TABLE 4:** The SPAN for negotiation preparation.

Opening offers	Issues			
	PE	DS	WP	AL
A^+^	16	8	2	DC
A^–^	20	12	1	LA

**TABLE 5:** The possible agreements.

Alternatives	Issues			
	PE	DS	WP	AL
A^+^	16	8	2	DC
A_1_	16	10	1.5	NY
A_2_	16	12	1	LA
A_3_	17	8	2	DC
A_4_	17	10	1.5	NY
A_5_	17	12	1	LA
A_6_	18	8	2	DC
A_7_	18	10	1.5	NY
A_8_	18	12	1	LA
A_9_	19	8	2	DC
A_10_	19	10	1.5	NY
A_11_	19	12	1	LA
A_12_	20	8	2	DC
A_13_	20	10	1.5	NY
A^–^	20	12	1	LA

“NY” in the rightmost column of Table 5 indicates that the NT has determined NY law to be a law option to consider during negotiation.

The NT uses Table 1 to assess the weights of each issue before employing Eq. 11 in assigning weights to each issue (Table 6).

**TABLE 6:** The weight of issues.

The NT members	Issues			
	PE	DS	WP	AL
NT_1_	(0.65, 0.80, 0.95)	(0.35, 0.50, 0.65)	(0.50, 0.65, 0.8)	(0.65, 0.80, 0.95)
NT_2_	(0.80, 1.00, 1.00)	(0.35, 0.50, 0.65)	(0.35, 0.5, 0.65)	(0.65, 0.80, 0.95)
NT_3_	(0.80, 1.00, 1.00)	(0.35, 0.50, 0.65)	(0.65, 0.8, 0.95)	(0.65, 0.80, 0.95)
NT_4_	(0.80, 1.00, 1.00)	(0.80, 1.00, 1.00)	(0.80, 1.00, 1.00)	(0.35, 0.50, 0.65)
NT_5_	(0.35, 0.50, 0.65)	(0.35, 0.50, 0.65)	(0.80, 1.00, 1.00)	(0.80, 1.00, 1.00)
Weight	(0.68, 0.86, 0.92)	(0.44, 0.60, 0.72)	(0.59, 0.76, 0.85)	(0.62, 0.78, 0.90)

Using Table 2, the NT converts the linguistic terms into fuzzy numbers to construct the fuzzy decision matrix (Table 7).

**TABLE 7:** The fuzzy decision matrix.

Alternatives	Issues			
	PE	DS	WP	AL
A^+^	16	8	2	(9, 10, 10)
A_1_	16	10	1.5	(3, 5, 7)
A_2_	16	12	1	(1, 3, 5)
A_3_	17	8	2	(9, 10, 10)
A_4_	17	10	1.5	(3, 5, 7)
A_5_	17	12	1	(1, 3, 5)
A_6_	18	8	2	(9, 10, 10)
A_7_	18	10	1.5	(3, 5, 7)
A_8_	18	12	1	(1, 3, 5)
A_9_	19	8	2	(9, 10, 10)
A_10_	19	10	1.5	(3, 5, 7)
A_11_	19	12	1	(1, 3, 5)
A_12_	20	8	2	(9, 10, 10)
A_13_	20	10	1.5	(3, 5, 7)
A^–^	20	12	1	(1, 3, 5)
Weight	(0.68, 0.86, 0.92)	(0.44, 0.60, 0.72)	(0.59, 0.76, 0.85)	(0.62, 0.78, 0.90)

Using Eqs 12, 24, 25, the NT constructs the normalized fuzzy decision matrix (Table 8).

**TABLE 8:** The normalized fuzzy decision matrix.

Alternatives	Issues			
	PE	DS	WP	AL
A^+^	1.00	1.00	1.00	(0.90, 1.00, 1.00)
A_1_	1.00	0.80	0.75	(0.30, 0.50, 0.70)
A_2_	1.00	0.67	0.50	(0.10, 0.30, 0.50)
A_3_	0.94	1.00	1.00	(0.90, 1.00, 1.00)
A_4_	0.94	0.80	0.75	(0.30, 0.50, 0.70)
A_5_	0.94	0.67	0.50	(0.10, 0.30, 0.50)
A_6_	0.89	1.00	1.00	(0.90, 1.00, 1.00)
A_7_	0.89	0.80	0.75	(0.30, 0.50, 0.70)
A_8_	0.89	0.67	0.50	(0.10, 0.30, 0.50)
A_9_	0.84	1.00	1.00	(0.90, 1.00, 1.00)
A_10_	0.84	0.80	0.75	(0.30, 0.50, 0.70)
A_11_	0.84	0.67	0.50	(0.10, 0.30, 0.50)
A_12_	0.80	1.00	1.00	(0.90, 1.00, 1.00)
A_13_	0.80	0.80	0.75	(0.30, 0.50, 0.70)
A^–^	0.80	0.67	0.50	(0.10, 0.30, 0.50)
Weight	(0.68, 0.86, 0.92)	(0.44, 0.60, 0.72)	(0.59, 0.76, 0.85)	(0.62, 0.78, 0.90)

Using Eq. 13, the NT constructs the weighted normalized fuzzy decision matrix (Table 9).

**TABLE 9:** The weighted normalized fuzzy decision matrix.

Alternatives	Issues			
	PE	DS	WP	AL
A^+^	(0.68, 0.86, 0.92)	(0.44, 0.60, 0.72)	(0.59, 0.76, 0.85)	(0.56, 0.78, 0.90)
A_1_	(0.68, 0.86, 0.92)	(0.35, 0.48, 0.58)	(0.44, 0.57, 0.64)	(0.19, 0.39, 0.63)
A_2_	(0.68, 0.86, 0.92)	(0.29, 0.40, 0.48)	(0.30, 0.38, 0.43)	(0.06, 0.23, 0.45)
A_3_	(0.64, 0.81, 0.87)	(0.44, 0.60, 0.72)	(0.59, 0.76, 0.85)	(0.56, 0.78, 0.90)
A_4_	(0.64, 0.81, 0.87)	(0.35, 0.48, 0.58)	(0.44, 0.57, 0.64)	(0.19, 0.39, 0.63)
A_5_	(0.64, 0.81, 0.87)	(0.29, 0.40, 0.48)	(0.30, 0.38, 0.43)	(0.06, 0.23, 0.45)
A_6_	(0.60, 0.76, 0.82)	(0.44, 0.60, 0.72)	(0.59, 0.76, 0.85)	(0.56, 0.78, 0.90)
A_7_	(0.60, 0.76, 0.82)	(0.35, 0.48, 0.58)	(0.44, 0.57, 0.64)	(0.19, 0.39, 0.63)
A_8_	(0.60, 0.76, 0.82)	(0.29, 0.40, 0.48)	(0.30, 0.38, 0.43)	(0.06, 0.23, 0.45)
A_9_	(0.57, 0.72, 0.77)	(0.44, 0.60, 0.72)	(0.59, 0.76, 0.85)	(0.56, 0.78, 0.90)
A_10_	(0.57, 0.72, 0.77)	(0.35, 0.48, 0.58)	(0.44, 0.57, 0.64)	(0.19, 0.39, 0.63)
A_11_	(0.57, 0.72, 0.77)	(0.29, 0.40, 0.48)	(0.30, 0.38, 0.43)	(0.06, 0.23, 0.45)
A_12_	(0.54, 0.69, 0.74)	(0.44, 0.60, 0.72)	(0.59, 0.76, 0.85)	(0.56, 0.78, 0.90)
A_13_	(0.54, 0.69, 0.74)	(0.35, 0.48, 0.58)	(0.44, 0.57, 0.64)	(0.19, 0.39, 0.63)
A^–^	(0.54, 0.69, 0.74)	(0.29, 0.40, 0.48)	(0.30, 0.38, 0.43)	(0.06, 0.23, 0.45)

Using Eqs 16–18, the NT calculates the separation measures, the $S_{i}^{+}\,\text{and}$$S_{i}^{-}$ of each alternative from the *A*^+^ and *A*^−^, and closeness coefficient $C\,C_{i}^{*}$ of each alternative, respectively (Table 10).

**TABLE 10:** Coefficient closeness of each alternative.

A_*i*_	A_1_	A_2_	A_3_	A_4_	A_5_	A_6_	A_7_	A_8_	A_9_	A_10_	A_11_	A_12_	A_13_
$S_{i}^{+}$	1.13	1.85	0.09	1.22	1.94	0.16	1.29	2.01	0.23	1.36	2.08	0.29	1.42
$S_{i}^{-}$	1.01	0.30	2.05	0.93	0.22	1.98	0.85	0.14	1.91	0.79	0.07	1.86	0.73
$C\,C_{i}^{*}$	0.47	0.14	0.96	0.43	0.10	0.92	0.40	0.07	0.89	0.37	0.03	0.87	0.34

**FIGURE 4:**
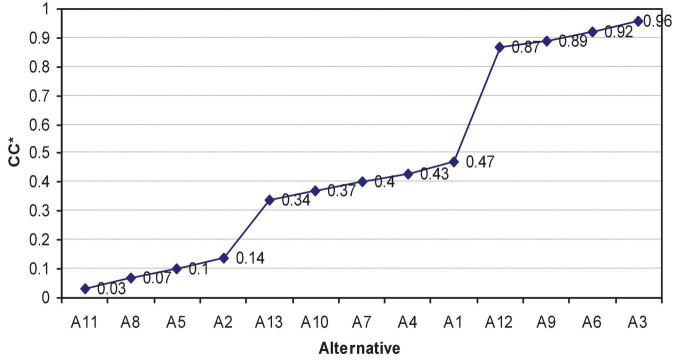
CC* value of the respective alternative.

Subsequently, the NT plots all CC* values in ascending order (Figure 4).

As presented in Figure 4, the NT considers various strategies, tactics, and concession methods (Table 11).

**TABLE 11:** Preparation of strategy, tactic, and concession methods.

Strategy	*ST*^1^ = [0.01,0.90] ={*A*_11_,*A*_8_,*A*_5_,*A*_2_,*A*_13_,*A*_10_,*A*_4_,*CPSTABLEENTERA*_7_,*A*_1_,*A*_12_,*A*_9_}*CPSTABLEENTERST*^2^ = [0.30, 0.70] ={*A*_13_,*A*_10_,*A*_4_,*A*_7_,*A*_1_}*CPSTABLEENTERST*^3^ = [0.40, 1.00] ={*A*_7_,*A*_4_,*A*_1_,*A*_12_,*A*_9_,*A*_6_,*A*_3_}
Tactic	Take-or-leave it, Bogey, Chinese crunch, Auction, Good guy-bad guy
Concession	0.01 incremental, 0.02 incremental, and 0.03 incremental

Here, *ST*^1^, *ST*^2^, and *ST*^3^ correspond to cooperative, hybrid, and distributive strategies, respectively. The incremental volume for concession is based on the CC* value.

By considering related factors in conjunction with the contents of Table 11, the NT determines several factors. First, this negotiation is based on *ST*^1^. Second, the incremental value for each concession is 0.03. Third, tactics should not be used. Fourth, the overall negotiating objective is *OO*^*v*^ (0.34, 0.43, 0.89). Fifth, other suppliers (with reference to the BATNA) should be used if the negotiation objectives cannot be reached.

Finally, the NT documents all information generated from the calculations in a negotiation plan, which is reviewed by all related stakeholders and then authorized for execution.

### Execution stage

**FIGURE 5:**
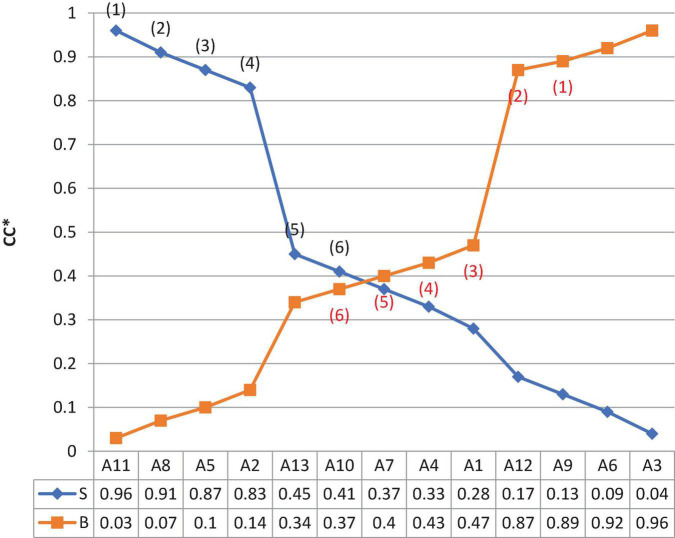
Record of negotiation.

The negotiation issues prepared by company B must first be confirmed by company S. The parties convene in the meeting room of company B. After a series of introductions, discussions, consultations, and concessions, an agreement is reached: alternative *A*_10_ = {19, 10, 1.5, NY}. The approaching path to agreement is graphically displayed in Figure 5.

During the negotiation, each party can only see their own graph.

After reaching an agreement, based on the negotiated price, the parties exchange the previously undisclosed information used for developing their individual negotiation plans. Based on the new information, the parties jointly discuss the weights assigned to each attribute (issue) and the ratings of each alternative under the price of 19 billion. With the new set of weights and alternatives, the proposed model is applied, and several alternatives preferable to the negotiated agreement are identified as *A_*new*_* = {19, 8, 2, DC}. The price is the same as that of *A*_10_, but company S has a shorter delivery period, and company B has a longer warranty period and a preferred set of applicable laws. The parties agree that this new package is more favorable than that initially agreed on. Thus, the negotiation concludes.

### Implementation stage

In this stage, the parties implement the agreement from the previous stage. However, 1 month before the delivery date, company B receives a letter from company S stating that because of a newly introduced technology, the control system specification requires modification, and the delivery schedule will therefore be delayed by 3 months. Company S is willing to reduce the price by 3% to compensate company B for possible losses from this scheduling delay. The two companies decide to settle these issues through negotiation, in which the proposed framework is employed once more.

## Conclusion

With the increasing use of project approaches around the world to handle business and deliver value, project success is important. Considering the gap between negotiation-related theory and practice in the project domain, and the fact that many PP negotiations do not work as expected; this study aims to develop a PP negotiation framework to help reduce the gap and better manage PP negotiations.

This study developed a practical framework by investigating studies from various knowledge areas and integrating relevant characteristics from widely employed models, namely the PPMP, TPNM, and fuzzy TOPSIS (Figure 2). The proposed framework has first used a team to identify the negotiation issues and corresponding ZOPAs. Next, all ZOPAs are used to establish a SPAN, and all possible agreements in the SPAN are evaluated to devise a negotiation plan from which quantitative values are assigned to all issues affecting the process or outcomes of negotiation in the early phase. Such quantitative values not only provide project negotiators with substantial information for preparing strategies, tactics, ways of concessions, and a BATNA but also enhance their capability and flexibility in adjusting and implementing agreements to attain win-win outcomes. The results of the numerical manipulation show that the proposed framework enables to help better manage PP negotiation in several ways.

First, PP negotiation is a work that supports the operation of the project and often faces the pressure of completion deadlines. This study proposed a systematic framework of three stages and sixteen steps (Figure 2), which can help the management to quickly master the negotiation work and make good planning and management. Negotiation experts agree that adequate preparation is the most difficult but the key to a successful negotiation; the proposed framework shows the same message (Figure 2). In practice, sound negotiation preparation requires an interdisciplinary team. The number of members, the professionalism of the members, the degree of cooperation of the members, and the leadership style of the team leader are all important details that affect the success or failure of negotiations and shall not be neglected.

Next, situations with incomplete information usually impede successful PP negotiation. In the present example, the framework employs two sets of data, the initial offers of the buyer and the seller, to evaluate and prioritize 13 possible agreements (Table 5), providing negotiators with a practical approach for handling insufficient information. The proposed framework can be used to evaluate more than several thousand possible agreements if required, providing negotiators with sufficient information for finding mutually satisfactory solutions.

Third, the preparation steps from “Collecting and Analyzing Information” to “Developing Strategies, Tactics, and Way of Concession” in the proposed framework (Figure 2) are designed to generate numerical values (Tables 3–11) and visualize information (Figure 4) using Fuzzy TOPSIS. The value generated can provide negotiators with quantitative references in understanding variables, making plans, and pursuing value. More importantly, some value of alternatives differs between sides but is equivalent in terms of CC* values (Table 10). Given that presenting a range of options for possible agreement is an essential principle for win-win negotiations. A set of equivalent possible agreements is particularly useful in concession making during the execution stage because they can increase the negotiation flexibility, resulting in a mutually satisfactory agreement.

Forth, under the execution stage (stage II) of the proposed framework, a reconciliation step is arranged after an agreement is reached (Figure 2). Through the exchange of all information used to develop their negotiation plans, a new agreement preferable to the initial one may emerge. A more favorable agreement can enhance cooperative relationships between involved parties, thus facilitating the implementation of the win-win PP negotiations. Several studies and publications have highlighted building long-term relationships as one of the most critical factors within and between organizations in the pursuit of project success and economic, social, and environmental sustainability (IPMA and ICB-OCB-IPMA, 2015; Trocki et al., 2020; Project Management Institute, 2021; Ladika and Budiman, 2022). In theory, using the negotiation framework proposed in this study could have positive effects beyond the project domain.

All methods and processes employed in the proposed framework are not only well established but also easy to implement. Because of its commonality, mutuality, and simplicity, the proposed framework can be generalized to other project negotiation settings to optimize project management regarding stakeholders, resources, and risks. Project negotiation is not an isolated event; rather, it proceeds under a systematic framework. All activities under that framework are aligned with the project objectives. For example, in PP negotiation, the negotiation is subordinate to the procurement, and the procurement is subordinate to the project. This systematic structure makes project negotiation a complex undertaking, requiring fuzzy set theory, decision-making methods, and negotiation models to evaluate variables and alternatives. Particularly, when the amount of information is insufficient.

The findings enable a systematic understanding of project negotiation regarding fuzzy decision-making and procurement negotiation. Interdisciplinary integration is a productive approach for closing the theory-practice gap in project negotiation settings. This study provides researchers and practitioners with a foundation to study refined models to enhance project negotiations with interdisciplinary integration.

Herein, the proposed framework was investigated through a numerical example. Studies can apply this framework to real-world cases to examine its function in a wide range of projects. The resulting comparisons can provide insight into the applicability of the proposed framework.

## Data availability statement

All data sets generated or analyzed for this study are presented in the article.

## Author contributions

CY collected and analyzed the data and wrote the initial draft. JL assisted with ideas, directions, and manuscript revisions. Both authors contributed to the article and approved the submitted version.
